# Maternal exposure to polychlorinated biphenyls and the secondary sex ratio: an occupational cohort study

**DOI:** 10.1186/1476-069X-10-20

**Published:** 2011-03-18

**Authors:** Carissa M Rocheleau, Stephen J Bertke, James A Deddens, Avima M Ruder, Christina C Lawson, Martha A Waters, Nancy B Hopf, Margaret A Riggs, Elizabeth A Whelan

**Affiliations:** 1 Division of Surveillance, Hazard Evaluations and Field Studies; Centers for Disease Control and Prevention, National Institute for Occupational Safety and Health; Cincinnati, Ohio, USA; 2 Department of Mathematical Science, University of Cincinnati, Cincinnati, Ohio, USA; 3 Division of Applied Research and Technology; Centers for Disease Control and Prevention, National Institute for Occupational Safety and Health; Cincinnati, Ohio, USA; 4 Institut universitaire romand de Santé au Travail/Institute for Work and Health (IST), Lausanne, Switzerland; 5 Coordinating Office for Terrorism, Preparedness and Emergency Response (Kentucky Department for Public Health); Centers for Disease Control and Prevention; Frankfort, Kentucky, USA

## Abstract

**Background:**

Though commercial production of polychlorinated biphenyls was banned in the United States in 1977, exposure continues due to their environmental persistence. Several studies have examined the association between environmental polychlorinated biphenyl exposure and modulations of the secondary sex ratio, with conflicting results.

**Objective:**

Our objective was to evaluate the association between maternal preconceptional occupational polychlorinated biphenyl exposure and the secondary sex ratio.

**Methods:**

We examined primipara singleton births of 2595 women, who worked in three capacitor plants at least one year during the period polychlorinated biphenyls were used. Cumulative estimated maternal occupational polychlorinated biphenyl exposure at the time of the infant's conception was calculated from plant-specific job-exposure matrices. A logistic regression analysis was used to evaluate the association between maternal polychlorinated biphenyl exposure and male sex at birth (yes/no).

**Results:**

Maternal body mass index at age 20, smoking status, and race did not vary between those occupationally exposed and those unexposed before the child's conception. Polychlorinated biphenyl-exposed mothers were, however, more likely to have used oral contraceptives and to have been older at the birth of their first child than non-occupationally exposed women. Among 1506 infants liveborn to polychlorinated biphenyl-exposed primiparous women, 49.8% were male; compared to 49.9% among those not exposed (n = 1089). Multivariate analyses controlling for mother's age and year of birth found no significant association between the odds of a male birth and mother's cumulative estimated polychlorinated biphenyl exposure to time of conception.

**Conclusions:**

Based on these data, we find no evidence of altered sex ratio among children born to primiparous polychlorinated biphenyl-exposed female workers.

## Background

Polychlorinated biphenyls (PCBs) are a group of synthetic aromatic chlorinated hydrocarbons that share a common biphenyl structure, but differ in the number and position of chlorine substitutions. PCBs were commercially produced in the United States from the 1920s until their ban in 1977. These compounds were used in lubricants, paints, adhesives, and sealants; as plasticizers in caulking compounds; and in the production of electrical capacitors and transformers. Over 30 years post-ban, workers can still be exposed to PCBs while maintaining or repairing older electrical equipment, or when remediating hazardous waste sites contaminated with PCBs [1]. PCBs continue to contaminate most lakes and rivers; recent data suggest that PCB concentrations may be rising in surface waters as glaciers melt, releasing their trapped PCBs [2]. PCBs also continue to be produced as an inadvertent byproduct of commercial paint and pigment production and weathering on outdoor surfaces [3,4]. PCBs also bioaccumulate and magnify in the food chain. Due to their persistence and pervasiveness, lipophilic PCBs are detectable in the adipose tissue of human populations throughout the world [1,5,6].

Multiple studies have demonstrated that PCBs can disrupt normal endocrine function in a variety of ways. Various PCB congeners have displayed the potential to alter the production of steroid hormones in human cells, thereby changing concentrations of estradiol, progesterone, testosterone, and cortisol [7]. PCBs may be estrogen agonists or estrogen antagonists depending on their structure [8,9]; various congeners can also affect activation of the androgen and glucocorticoid receptors [10,11]. PCBs may also impact thyroid homeostasis [12,13]. Because of their potential to disrupt normal endocrine function, it has been hypothesized that parental exposures to PCBs could be associated with a reduction in the ratio of male to female live births, called the secondary sex ratio [14,15]. It has previously been hypothesized that parental hormone concentration at the time of conception can impact the likelihood of survival or fertilization by male sperm, or the viability of a male zygote, through hormone-mediated processes [16-18]. The secondary sex ratio fluctuates slightly over time and between countries; historically, about 51.2% of liveborn infants in the United States are male [19]. The most consistent characteristic associated with the secondary sex ratio is birth order [19-23]. Other, less consistent associations include maternal and paternal age [20,22,24,25], parental education and race [24], frequency or timing of coitus [26], and certain infections [27-29].

Epidemiological studies examining deficits in male births born to parents exposed to PCBs have shown mixed results. Studies of the Yucheng, Taiwan, PCB poisoning incident showed no change in the secondary sex ratio of children (n = 902) born to PCB-exposed women, but a significant decrease among children (n = 286) born to men exposed to PCBs prior to age 20 [30]. Yoshimura and colleagues also found no significant change in the secondary sex ratio following the 1968 Yusho, Japan, poisoning incident in which thousands of people ingested rice oil contaminated with PCBs [31]. The small number of births (n = 85) in that study, however, precluded stratification of results by parent (mother versus father) or age at which exposure occurred in the parent. Similarly, studies in populations which consumed high amounts of PCB-contaminated fish have not produced consistent associations between secondary sex ratio and parental serum PCB concentrations [32,33].

Potential adverse human health effects from exposure to PCBs may be identified most readily in groups with the greatest potential exposures: worker populations. To examine whether elevated PCB concentrations are associated with changes in the secondary sex ratio, we examined maternal preconceptional PCB exposure in a large cohort of women who worked at electrical capacitor producing plants in Indiana, Massachusetts and New York. All three plants used multiple commercial PCB mixtures containing 41-54% chlorine: in Indiana, primarily Aroclor 1242 from 1957-1971 and Aroclor 1016 from 1971-1977 [34]; in New York, Aroclor 1254 (phased out in the 1950's), Aroclor 1242, and Aroclor 1016 (first used in 1971); and in Massachusetts, Aroclor 1254, 1242, and 1016 as well (dates unknown) [35]. To date, this is the largest study to examine secondary sex ratios among first births to an occupationally PCB-exposed maternal population.

## Methods

### Study population

The study population was drawn from a subset of the 14,178 women included in prior retrospective cohort mortality studies of workers at three electrical capacitor manufacturing plants located in New York (4,855 women), Massachusetts (8,465 women), and Indiana (857 women). To be included in our study population, these women must have worked for at least one year at any of the three capacitor plants (5,752 women), completed a questionnaire about their reproductive history (3,952 women), and had given birth to a live infant (2,639 women).

The National Institute for Occupational Safety and Health (NIOSH) initially collected demographic and work history data from the three plants in the 1970s [35,36]. These cohorts were updated periodically for use in multiple NIOSH studies [37-43]. Data on workers from all three plants were recently updated using new job-exposure matrices (JEMs) to estimate exposures [34,44-46]; these data were used in mortality and incidence studies of PCB-exposed workers [39-43]. These plant-specific semi-quantitative JEMs incorporated detailed information on the processes at each plant, floorplans and descriptions of the plants (including changes over time), a small number of air samples, information about historical process changes, and job descriptions within the plants. Factors that could affect PCB exposure (exposure determinants, such as plant location or specific tasks) were identified for all unique jobs. Jobs with similar exposure determinants were combined into categories, which were rated qualitatively for intensity and frequency of PCB exposures via both inhalation and dermal exposure routes [34,44,46]. The final exposure rating combined both dermal and inhalation exposure values. Plant-specific air concentrations were used to anchor the PCB exposure rating, and may thus be combined across plants.

Along with the cohort and JEM updates, an interview study of cancer and reproductive history was conducted among the 5,752 women who worked for at least one year at any of the three capacitor plants; these methods have been described in detail previously [43]. Briefly, addresses and telephone numbers were updated and a self-administered questionnaire was mailed to all women or their next-of-kin (25% of the cohort had died) for whom an accurate address could be found (n = 4,564, 79% of the study population). After two mailings and a reminder postcard, non-respondents were telephoned to complete the questionnaire. Overall, completed questionnaires were received for 3,952 women (87%). The questionnaire was translated into Spanish and Portuguese due to the ethnic make-up of the cohort, and was approved by the NIOSH Human Subjects Review Board.

The questionnaire collected data on relevant non-occupational risk factors such as medical and reproductive history (including number, dates, and outcomes of pregnancies), race and ethnicity, education, height, weight, smoking, and family medical history. Respondents selecting "other" for race were prompted to further specify their race/ethnicity(s). While most workers were white, the Massachusetts plant included a number of workers of Cape Verdean origin [40]; most Cape Verdeans are of mixed (maternal) African and (paternal) Portuguese ancestry. Self-identified race may have been inconsistent among these workers since neither "Cape Verdean" nor "biracial" were listed among possible responses for race/ethnicity.

In the reproductive history section of the questionnaire, a respondent (or her proxy) was asked if she had ever been pregnant; if so, how many times she had been pregnant (including live births, stillbirths, miscarriages, abortions, and tubal pregnancies). For each reported pregnancy, we solicited the pregnancy end date; how long the pregnancy lasted (1-3 months, 4-6 months, 7-9 months); whether the pregnancy was single or multiple; whether the pregnancy ended in a live birth, stillbirth, miscarriage, induced abortion, or other; and the sex of each live born infant.

### Exposure variables and covariates

Cumulative PCB exposure to the time of the index conception was calculated from the plant-specific JEMs. Job categories were rated qualitatively for intensity (baseline, low, medium, high) and frequency (continuous, intermittent) of PCB exposures. Inhalation and dermal exposure were rated separately for each category. Inhalation intensity scores were quantitatively mapped based on air PCB measurements in the plant. No dermal measurements were available, and thus the dermal ratings are unitless. For both dermal and inhalation exposure, the products of the intensity and frequency ratings were calculated; the dermal and inhalation exposure values were then averaged together as a final value for each plant-specific exposure category (and thereby for each plant-specific job within that category) [34,44,46].

For the purposes of our analysis, women with only background levels of occupational PCB exposure at the time of conception were considered unexposed; these women may have worked in an area of the plant where no PCBs were ever used prior to conceiving the child, or may not have worked in the plant until after the conception. Cumulative occupational exposure to PCBs to the time of conception was modeled as a bivariate (any/none), categorical (quartiles of exposure vs. none), and continuous variable. Births occurring prior to the earliest date at which PCBs were used in a given plant were excluded to prevent confounding by time period, since all these early births would be non-occupationally exposed by definition.

To avoid confounding by birth order or pregnancy-related changes in BMI and PCB body burden[47], the analysis was restricted to liveborn singleton births from primiparous women. Very preterm births (occurring at less than seven months gestation) were excluded (n = 38) from the main analysis for both consistency with other studies and to prevent confounding[48-50], since the sample size was too small for adjustment; including these subjects in the analysis, however, did not alter our results (data not shown). Women reporting a BMI below 15 at age 20 were also excluded (n = 6), since this is below the threshold for starvation and we were unable to distinguish between extreme values that actually existed, which could create confounding by nutritional status, and those that were due to reporting errors. This left 2,595 women and their infants available for analysis.

Potential covariates evaluated for inclusion in the final multivariate model included BMI at age 20 (< 18.5, 18.5 - 21.9, 22-24.9, 25-29.9, 30+), highest education level attained (grade school only, some high school, completed high school or GED, some college or professional training, college degree or higher), race (white, nonwhite or mixed race), use of oral contraceptives in the year prior to the conception (yes, no), smoking prior to giving birth to the index child (ever/never), plant (Massachusetts, Indiana, New York), and whether the questionnaire was completed by the worker or a proxy. Maternal age at time of conception was evaluated as both a continuous and categorical (≤ 18, 19-24, 25-29, ≥ 30) variable. To assess cohort effects, year of mother's birth and year of infant's birth were assessed as both continuous and categorical variables. Among workers in the Massachusetts plant, we also stratified our analysis by exposure occurring during U.S. involvement in World War II (1941-1945) (any PCB exposure in 1941-1945 versus no exposure during 1941-1945), when production was increased and the plants were primarily staffed by women. A sub-analysis also examined outcomes for those with Portuguese last names (as an approximate surrogate for Cape Verdean ethnicity) to better assess differences between this group and the remainder of the study population.

### Statistical analysis

Continuous variables were modeled as linear, log-transformed, and quadratic variables; then evaluated as categorical variables as described previously. The Akaike Information Criterion (AIC) [51] was used to select variables that provided the best fit to the data. Bivariate analyses were conducted to identify potential confounding variables using either the Chi-square test for categorical variables or linear regression for continuous variables. Variables that were significantly related to both the secondary sex ratio and PCB exposure terms (α ≤ 0.10) or that changed the odds ratio estimate for the main effect (≥ 10%), were included in multivariate model selection. Logistic regression models were created via a hybrid backward selection scheme. The AIC was used to select the most parsimonious model providing an adequate fit to the data. All analyses were conducted using SAS 9.1.3 (SAS Institute, Cary, NC). Stratified analyses examined whether respondent (woman or a proxy), age at conception (younger than age 20, age 20 or older), or plant (Indiana, Massachusetts, New York) impacted results.

## Results

Data from 2,595 singleton live births of primiparous female workers were analyzed. The percentage of primiparous workers with preconceptional PCB exposure varied by plant, from 41% in the New York plant to 69.8% in the Massachusetts plant (Table 1). Workers in the Massachusetts plant were the most highly exposed. The Massachusetts plant also opened 10 years prior to the New York plant and 19 years prior to the Indiana plant (note that the Massachusetts plant was operating during World War II, when production demands were high to support war efforts). Women had been employed in the plants for an average of 2-2.6 years prior to conceiving their first liveborn child. Primiparous workers in the Massachusetts plant cohort tended to have lower education levels than workers in the New York and Indiana plant cohorts, and were more likely to be non-white.

**Table 1:** Characteristics of primipara, by plant

		Indiana Plant (n = 173)	Massachusetts Plant (n = 1481)	New York Plant (n = 941)
**PCB production years ^a^**		1957-1977	1938-1977	1948-1977

**Estimated cumulative occupational PCB exposure, to estimated date of conception ^b^**				

	Median	4,868	193,320	20,988

	Interquartile range	2,460-8,510	84,672-397,512	8,700-54,120

**Duration of employment (years), prior to estimated date of conception ^c^**				

	Median	2.0	2.6	2.0

	Interquartile range	1.1-2.7	1.3-4.6	1.1-3.5

**Occupationally PCB exposed prior to estimated date of conception (n, %)**		86 (49.7%)	1,034 (69.8%)	386 (41.0%)

**Reproductive history reported by proxy**		2 (1.2%)	190 (12.8%)	82 (8.7%)

**Gender of offspring (n, %)**				

	Males	85 (49.1%)	724 (48.9%)	484 (51.4%)

	Females	88 (50.9%)	757 (51.1%)	457 (48.6%)

**Maternal race/ethnicitiy (n, %)**				

	White, non-Hispanic	165 (95.4%)	1,234 (83.3%)	914 (97.1%)

	Non-white or multiracial	8 (4.6%)	247 (16.7%)	27 (2.9%)

**Maternal education (n, %)**				

	Grade school only	1 (0.6%)	125 (8.6%)	7 (0.8%)

	Some high school	9 (5.2%)	477 (32.7%)	98 (10.5%)

	High school graduate or GED	69 (39.9%)	522 (35.8%)	537(57.3%)

	Some college or professional training	75 (43.4%)	261 (17.9%)	229(24.4%)

	College degree or higher	19 (11%)	74 (5.1%)	67 (7.1%)

**Maternal Age (n, %)**				

	18 or younger	30 (17.3%)	141 (9.5%)	147 (15.6%)

	19-24	100 (57.8%)	910 (61.4%)	550 (58.5%)

	25-29	29 (16.8%)	286 (19.3%)	156 (16.6%)

	30 or older	14 (8.1%)	144 (9.7%)	88 (9.4%)

IR = interquartile range

^a ^Births occurring prior to the earliest dates of PCB production at each plant were excluded

^b ^Cumulative exposure was estimated using the combined inhalation-dermal job exposure matrix

^c ^Employment refers to time worked while PCBs were in use at the plants.

Fifty-eight percent (n = 1506) of participants were exposed to PCBs prior to conceiving their first liveborn children. Among these, 750 (49.8%) had male infants compared to 543 (49.9%) male infants among women not exposed to PCBs preconceptionally. Table 2 shows the sex distribution of offspring, stratified by exposure status, according to demographic characteristics of the mother. The gender distribution of offspring was generally comparable between exposed and unexposed workers in terms of education, smoking history, race, and BMI at age 20. Preconceptionally exposed workers tended to be older at the time they conceived their first liveborn child, compared to preconceptionally unexposed workers. Exposed workers were also more likely to have used oral contraceptives in the year prior to conceiving their first child, though this difference was not statistically significant.

**Table 2:** Gender distribution of first live-born offspring, by maternal characteristics and exposure status.

		PCB Exposed		PCB Unexposed	
	
		Male child N (%)	Female child N (%)	Male child N (%)	Female child N (%)
	
		750 (49.8)	756 (50.2)	543 (49.9)	546 (50.1)
**Maternal Education**					

	Grade school only	35 (52.2)	32 (47.8)	30 (45.5)	36 (54.6)

	Some high school	167 (49.1)	173 (50.9)	120 (49.2)	124 (50.8)

	High school graduate or GED	308 (51.3)	292 (48.7)	262 (49.6)	266 (50.4)

	Some college or professional training	163 (45.0)	199 (55.0)	104 (51.2)	99 (48.8)

	College degree or higher	70 (57.4)	52 (42.6)	24 (63.2)	14 (36.8)

**Oral contraceptive use in year prior to conception**					

	Yes	76 (54.3)	64 (45.7)	14 (53.9)	12 (46.2)

	No	674 (49.3)	692 (50.7)	529 (49.8)	534 (50.2)

**Maternal cigarette consumption prior to conception**					

	Yes	295 (51.0)	283 (49.0)	188 (49.9)	189 (50.1)

	No	455 (49.0)	473 (51.0)	355 (49.9)	357 (50.1)

**Maternal race/Ethnicity**					

	White, non-Hispanic	684 (50.0)	684 (50.0)	478 (49.4)	490 (50.6)

	Other	66 (47.8)	72 (52.2)	65 (53.7)	56 (46.3)

**Maternal age at conception**					

	18 or younger	35 (54.7)	29 (45.3)	147 (57.9)	107 (42.1)

	19-24	420 (47.7)	460 (52.3)	317(46.6)	363 (53.4)

	25-29	189 (52.2)	173 (47.8)	65 (59.6)	44 (40.4)

	30 or older	106 (53.0)	94 (47.0)	14 (30.4)	32 (69.6)

**Maternal BMI at age 20**					

	15.0 to < 18.5	112 (52.1)	103 (47.9)	75 (53.6)	65 (46.4)

	18.5 to < 22.0	382 (50.7)	372 (49.3)	261 (48.2)	280 (51.8)

	22.0 to < 25.0	135 (44.9)	166 (55.2)	100 (46.7)	114 (53.3)

	25.0 to < 30.0	44 (50.0)	44 (50.0)	39 (59.1)	27 (40.9)

	30.0+	18 (58.1)	13 (41.9)	7 (46.7)	8 (53.3)

Multivariate analyses controlling for mother's age and year of birth found no significant association between the odds of a male birth and mother's cumulative PCB exposure at time of conception, regardless of whether exposure was modeled as a continuous or categorical variable (Table 3). The sex ratio of offspring did not vary according to whether the questionnaire was completed by the worker or a proxy, or whether Portuguese last name was included as a covariate (data not shown). Stratifying the analysis by age at conception (younger than age 20, age 20 or older) did not affect the relationship between maternal occupational PCB exposure and sex ratio of offspring (data not shown). Neither adjusting for plant in the main analysis nor conducting a separate analysis within each plant (Indiana, Massachusetts, New York) affected the relationship between maternal occupational PCB exposure and the secondary sex ratio (see additional file 1: Odds of birth of a male infant among women occupationally PCB-exposed and unexposed prior to estimated date of conception, adjusted for plant, mother's date of birth, and maternal age at the birth of her first live-born child). There was also no trend toward an increasing association across increasing categories of preconceptional exposure (versus no exposure) and odds of a male birth. The percentage of male infants born to workers in the Indiana and Massachusetts plants was slightly lower (~49% male) than that observed among workers in the New York plant, however this percentage was similar between the children of occupationally exposed versus occupationally unexposed mothers (Table 4). There was also no difference in the relationship between preconceptional PCB exposure and the secondary sex ratio of workers in the Massachusetts plant when we examined PCB exposure occurring in 1941-1945 (World War II) compared to other time periods (data not shown).

**Table 3:** Crude and adjusted odds of birth of a male infant among women occupationally PCB-exposed and unexposed prior to estimated date of conception

Estimated Cumulative PCB exposure^a^, at estimated date of conception		Exposed Women (n)	Crude OR (95% CI)	Adjusted^b^ OR (95% CI)
Continuous model (log linear)				

	Per 100,000 increase	1,506	1.00 (0.98, 1.03)	1.01 (0.98, 1.03)

				

Categorical model				

	No exposure	1,089	1.00 (ref)	1.00 (ref)

	> 0 to <27,900	377	0.94 (0.75, 1.19)	0.93 (0.73, 1.19)

	27,900 to <108,140	376	1.14 (0.90, 1.44)	1.11 (0.87, 1.41)

	108,140 to <300,216	377	0.90 (0.72, 1.14)	0.90 (0.71, 1.15)

	300,216+	376	1.02 (0.81, 1.29)	1.06 (0.83, 1.36)

^a ^Cumulative exposure was estimated using the combined inhalation-dermal job exposure matrix

**^b ^**Adjusted for the mother's date of birth, and age at birth of her first born

**Table 4:** Crude and adjusted odds of birth of a male infant among women occupationally PCB-exposed and unexposed women prior to conception, stratified by plant

**Estimated Cumulative PCB exposure ^a^**, at estimated date of conception		Women (n)	Crude OR (95% CI)	Adjusted^b^ OR (95% CI)
**Indiana Plant (n = 173)**				

	No exposure	87	1.00 (ref)	1.00 (ref)

	> 0 to <2,460	21	0.98 (0.37, 2.55)	1.19 (0.43, 3.28)

	2,460 to <4,867.5	22	1.07 (0.42, 2.74)	1.20 (0.42, 3.39)

	4,867.5 to <8,510	21	0.36 (0.13, 1.01)	0.39 (0.12, 1.25)

	8,510+	22	0.74 (0.29, 1.90)	0.81 (0.27, 2.42)

**Massachusetts Plant (n = 1481)**				

	No exposure	447	1.00 (ref)	1.00 (ref)

	> 0 to <84,672	258	1.06 (0.78, 1.44)	1.06 (0.77, 1.47)

	84,672 to <193,320	258	1.08 (0.79, 1.47)	1.09 (0.78, 1.52)

	193,320 to <397,512	259	0.90 (0.67, 1.23)	0.93 (0.67, 1.29)

	397,512+	259	1.16 (0.85, 1.57)	1.16 (0.84, 1.61)

**New York Plant (n = 941)**				

	No exposure	555	1.00 (ref)	1.00 (ref)

	> 0 to <8,700	96	0.85 (0.55, 1.32)	0.85 (0.54, 1.34)

	8,700 to <20,988	97	1.07 (0.70, 1.65)	1.08 (0.69, 1.71)

	20,988 to <54,120	96	1.14 (0.74, 1.77)	1.07 (0.66, 1.73)

	54,120+	97	1.22 (0.79, 1.88)	1.20 (0.74, 1.96)

^a ^Cumulative exposure was estimated using the combined inhalation-dermal job exposure matrix

**^b ^**Adjusted for the mother's date of birth and maternal age at the birth of her first live-born child

## Discussion

The strengths of our study include restricting to primiparous births to prevent confounding by birth order, a large sample size, detailed plant and time-specific JEMs grounded by air monitoring samples and corroborated with serum samples, and evaluating an occupational cohort with much higher PCB exposure than previous population-based samples. We did not find an association between cumulative maternal PCB exposure prior to conception and infant sex ratio among female primiparous workers in three electrical capacitor plants. This finding is consistent with a recent population-based study of parental PCB blood concentrations and offspring gender, which also noted no increase in the proportion of males born to mothers with the highest category of PCB exposure [52]. A small increase in male births was suggested in that study, however, when both mothers and fathers were exposed to the highest category of PCBs--suggesting that paternal PCB exposure might be associated with the secondary sex ratio [52].

Although our results conflict with two other recent studies of blood PCB concentration and secondary sex ratio, the discrepancy between these studies and ours may be explained, in part, by variations in study design and population. Hertz-Picciotto and colleagues reported a 7% decrease in the sex ratio of offspring per 1 ppb increase in PCB burden in a population-based study [53], and Weisskopf and colleagues reported an 80% reduction in the secondary sex ratio among mothers in the highest quintile of PCB exposure from sport-caught fish [33]. Both of these studies used one-time serum PCB measurements which tend to only capture PCBs with long half-lives, whereas our plant-specific JEMs were able to account for long-term patterns of PCB exposure.

Our study also restricted the analysis to primiparous births (to n = 2595 mothers), thereby eliminating both potential confounding and effect modification by birth order, pregnancy-related changes in BMI, or breast-feeding. Previous studies have indicated an association between sex ratio of infants and their birth order [21,22,24]. Pregnancy-associated changes in BMI, and increased PCB clearance through breast-feeding, could result in associations between birth order and blood PCB concentrations. Hertz-Picciotto and colleagues did not adjust or stratify by birth order, possibly due to limitations in the sample size (n = 399 mothers) [53]. Weisskopf and colleagues adjusted for parity and the presence of an older male sibling, but because of their small sample size (n = 173 mothers) were not able to examine the potential for effect modification by parity and BMI [33].

Additionally, the route of PCB exposure varied across all these studies, which could impact absorption and metabolism of congeners. In our study, most exposure came from dermal absorption or inhalation of PCBs in an occupational setting; in the study by Weisskopf and colleagues, exposure primarily occurred through ingestion of sport-caught fish; and in the study by Hertz-Picciotto and colleagues, environmental exposure likely occurred through both ingestion and inhalation routes.

Perhaps the most important factor distinguishing our study from these previous studies, however, is that the magnitude of exposure in those study populations was substantially lower than in our occupationally exposed group. Weisskopf and associates reported a mean serum PCB concentration of 10.7 μg/L for mothers in the highest quintile of exposure [33], while Hertz-Picciotto and associates reported a mean serum PCB concentration of 1.9 μg/L for mothers in the highest decile of exposure [53]. By contrast, a study of the New York plant in 1976 found that workers (both male and female) in areas with high air PCB concentrations had serum L-PCB concentrations of 100 μg/L (geometric mean); female workers overall had serum H-PCB concentrations of 8 μg/L (geometric mean) [54]. Average serum values were 502 μg/L for lower chlorinated PCBs (L-PCBs) and 44 μg/L for higher chlorinated PCBs (H-PCBs) among a sample of current workers in 1977 in the capacitor-production area of the Indiana plant, and 237 μg/L L-PCBs and 51 μg/L H-PCBs among workers in the maintenance areas of that plant [55]. The PCB-exposed workers in our cohort therefore experienced PCB exposures substantially higher than the environmentally exposed individuals in other studies of maternal PCB exposure and secondary sex ratio. If a true association existed between PCB exposure and secondary sex ratio, we would expect to see that risk magnified in more highly exposed populations, such as we studied.

Additionally, no association between high-dose PCB exposure and secondary sex ratio has been observed in accidental contamination events. Rogan and colleagues found no change in the sex ratio of children born to women exposed to PCBs and polychlorinated dibenzofurans (PCDFs) from contaminated cooking oil in Taiwan in the 1978-79 Yucheng incident [56], despite mean serum PCB concentrations of 49.3 μg/L [57]. A significant decrease in the secondary sex ratio was later noted, however, among children born to exposed men - provided the men had been aged less than 20 at the time of exposure [30]. It was postulated that the Yucheng exposure could have caused damage to the young men's developing reproductive systems. Similarly, an analysis of maternal PCB and PCDF exposure from the Yusho incident did not note a decrease in male births; however, the small number of births in this population precluded a detailed analysis [31]. These studies might not be directly comparable with ours, however, due to their high accidental one-time exposures including both PCBs and PCDFs, with exposure occurring through ingestion; as opposed to this study, where the workers had long-term dermal and inhalation exposure.

PCBs have been suggested as a cause of altered secondary sex ratios due to their potential to disrupt normal endocrine function. Even the hypothesis that endocrine disruption could result in alterations in the secondary sex ratio, however, has inconsistent support in the scientific literature. Studies of children and grandchildren of women exposed to diethylstilbestrol (DES), an extremely potent endocrine disruptor, have failed to show perturbations in the proportion of male births [58,59]. Other data show that the majority of PCB transfer from mothers to their offspring occurs during breastfeeding, not *in utero *[60].

Besides consistency with other studies of high-dose PCB exposure and the ability to limit analysis to primiparous births, our study benefits from several unique strengths. The sample size is large, allowing us to have adequate power to detect associations. The estimated cumulative preconceptional PCB dose among exposed workers in our study covered a wider range than has been analyzed elsewhere, allowing us to examine whether a threshold effect or dose-response relationship existed. This study used JEMs to assign PCB exposure to workers at three electrical capacitor plants. Using a JEM, rather than direct monitoring for all workers, may introduce some exposure misclassification that would most likely dilute any observed association. Our JEMs, however, were based on industrial hygienist visits to the plants in question, detailed diagrams of the plant layout and processes, air samples, and some individual monitoring results. Using this sort of detailed information to inform a JEM improves the accuracy of exposure assessment [61]. Though we relied on proxy respondents to complete questionnaires when workers were deceased, the outcome under study (gender of liveborn children) is not likely subject to misreporting as it is discrete and would be known to a proxy respondent. Exposure metrics did not rely on self-report, but on plant records.

Despite these strengths, our results are subject to several limitations. First is our inability to fully distinguish between types of PCBs that workers were exposed to. Though offspring of workers in the New York plant had a reversed sex ratio versus that observed in Indiana and Massachusetts, the PCB mixtures used in New York were similar to those used in Massachusetts; a more detailed analysis by type of PCB, however, cannot be performed based on these data.

Another limitation of our study is the potential for exposure misclassification of workers. A number of personal and workplace factors which we were unable to account for may influence worker exposure to PCBs. Most of the women in our study-whether occupationally exposed or not-- also lived near the factories where they worked, and may have had additional PCB exposure from environmental contamination; this further increases potential misclassification of PCB exposure. Repeated serum PCB measurements would have provided more accurate measures of absorbed PCB dose from all sources, but were only available from a small number of workers and not at the time of conception. In small biomonitoring studies at the Indiana and New York plants, however, serum PCB concentrations of exposed workers were 8-50 times higher than those of unexposed workers or people living in the surrounding communities for H-PCBs, and two to four times higher for L-PCBs [54,55]. Serum PCB concentrations correlated fairly well with cumulative exposure estimates (combining both inhalation and dermal exposure) from the JEM in a sample of workers in the Indiana plant [34]; this suggests that our results cannot be simply attributed to misclassification.

Because most of the women in our study were also exposed to PCBs by living in the communities around the plants, our study might have failed to detect a true relationship - but only if PCBs exerted a threshold (rather than dose-response) effect on secondary sex ratios at doses similar to environmental levels. We observed that the percentages of male infants born to workers in the Indiana and Massachusetts plants were slightly lower than the national average (~49% compared to ~51% for the same time periods), but the secondary sex ratio was similar between occupationally exposed and unexposed workers. The sex ratio observed in the New York plant, however, was similar to the national averages. This argues against a threshold effect on the secondary sex ratio caused by environmental exposure to PCBs among those who were not exposed to PCBs at work.

Unmeasured paternal exposure to PCBs might have contributed to the lower rates of male births we observed among mothers working in the Indiana and Massachusetts plant, which previous studies suggest might be related to the secondary sex ratio [30,52,62]. Some fathers may have also worked in one of these plants, and could have been highly exposed to PCBs. Fathers living near the Indiana and Massachusetts plants might also have had higher environmental exposure to PCBs than fathers living near the New York plant. Unfortunately, we were unable to account for any paternal PCB exposure. Our finding of no association between maternal PCB exposure and the secondary sex ratio does not rule out an association between paternal PCB exposure, or combined parental PCB exposure, and the secondary sex ratio. We were also unable to obtain information on paternal age, which has been linked to the secondary sex ratio in previous studies.

## Conclusions

Overall, our finding of no association between maternal occupational PCB exposure and sex ratio of offspring is consistent with other studies of presumably high-dose PCB exposure. This is one of the largest cohort studies of PCB-exposed female workers to date, and our results are strengthened by our ability to account for occupational PCB exposure over time, restriction of our study set to primiparous live births, and high levels of PCB exposure among those exposed at the time of the index conception. Despite some limitations, a lack of association between highly PCB-exposed women and altered sex ratios in their offspring suggests that no association will exist for women in the general population with generally lower PCB exposure. We were unable to examine potential associations between PCBs and other reproductive outcomes, such as infertility, spontaneous abortion, and stillbirth; future work should explore the competing risks of these outcomes.

## Abbreviations

AIC: Akaike Information Criterion; JEM: job-exposure matrix; PCB: polychlorinated biphenyl; L-PCB: lower chlorinated polychlorinated biphenyl; H-PCB: higher chlorinated polychlorinated biphenyl; DES: diethylstilbestrol; BMI: body mass index; NIOSH: National Institute for Occupational Safety and Health; GED: General Education Diploma; PCDF: polychlorinated dibenzofurans; ppb: parts per billion.

## Competing interests

The authors declare that they have no competing interests.

## Authors' contributions

The work presented here was carried out in collaboration between all authors. CR coordinated the study, assisted in interpreting results, and drafted the manuscript. SB and JD conducted statistical analysis and interpretation. CL assisted in coordination of the study and writing the manuscript. AR conceived of the study, provided expertise in the area of secondary sex ratios, assisted in interpretation of results, and assisted in writing the manuscript. MR coordinated preliminary analysis, and assisted in drafting the manuscript. MW and NH provided technical expertise regarding the industrial hygiene assessment. EW assisted in coordination of the study and revising the manuscript. All authors have contributed to, read, and approved the final manuscript.

## Supplementary Material

Additional file 1 **Odds of birth of a male infant among women occupationally PCB-exposed and unexposed prior to estimated date of conception, adjusted by plant**. This table (adjusted_by_plant.doc), in Microsoft Word 2003, displays the odds of birth of a male infant among women occupationally PCB-exposed and unexposed prior to estimated date of conception, adjusted for plant, mother's date of birth, and maternal age at the birth of her first live-born child. Click here for file

## References

[B1] Agency for Toxic Substances and Disease Register (ATSDR) Toxicological profile for polychlorinated biphenyls (update). Atlanta 2000 36888731

[B2] Carrie J Wang F Sanei H Macdonald RW Outridge PM Stern GA Increasing contaminant burdens in an arctic fish, Burbot (Lota lota), in a warming climate Environ Sci Technol 2009 44 316 322 10.1021/es902582y 19957995

[B3] Hu D Hornbuckle KC Inadvertent polychlorinated biphenyls in commercial paint pigments Environ Sci Technol 2009 44 2822 2827 10.1021/es902413k PMC2853905 19957996

[B4] Rodenburg LA Guo J Du S Cavallo GJ Evidence for unique and ubiquitous environmental sources of 3,3'-dichlorobiphenyl (PCB 11) Environ Sci Technol 2010 44 2816 2821 10.1021/es901155h 20384375

[B5] Centers for Disease Control and Prevention (CDC) Fourth National Report on Human Exposure to Environmental Chemicals. Atlanta 2003

[B6] World Health Organization Polychlorinated Biphenyls and Terphenyls, Geneva 1993 2 [Environmental Health Criteria, No. 140]

[B7] Kraugerud M Zimmer KE Dahl E Berg V Olsaker I Farstad W Ropstad E Verhaegen S Three structurally different polychlorinated biphenyl congeners (Pcb 118, 153, and 126) affect hormone production and gene expression in the human H295R in vitro model J Toxicol Environ Health A 2010 73 1122 1132 10.1080/15287394.2010.484338 20574914

[B8] Bonefeld-Jorgensen EC Andersen HR Rasmussen TH Vinggaard AM Effect of highly bioaccumulated polychlorinated biphenyl congeners on estrogen and androgen receptor activity Toxicology 2001 158 141 153 10.1016/S0300-483X(00)00368-1 11275356

[B9] Connor K Ramamoorthy K Moore M Mustain M Chen I Safe S Zacharewski T Gillesby B Joyeux A Balaguer P Hydroxylated polychlorinated biphenyls (PCBs) as estrogens and antiestrogens: structure-activity relationships Toxicol Appl Pharmacol 1997 145 111 123 10.1006/taap.1997.8169 9221830

[B10] Portigal CL Cowell SP Fedoruk MN Butler CM Rennie PS Nelson CC Polychlorinated biphenyls interfere with androgen-induced transcriptional activation and hormone binding Toxicol Appl Pharmacol 2002 179 185 194 10.1006/taap.2002.9371 11906248

[B11] Schrader TJ Cooke GM Effects of Aroclors and individual PCB congeners on activation of the human androgen receptor in vitro Reprod Toxicol 2003 17 15 23 10.1016/S0890-6238(02)00076-X 12507654

[B12] Hagmar L Polychlorinated biphenyls and thyroid status in humans: a review Thyroid 2003 13 1021 1028 10.1089/105072503770867192 14651786

[B13] Salay E Garabrant D Polychlorinated biphenyls and thyroid hormones in adults: a systematic review appraisal of epidemiological studies Chemosphere 2009 74 1413 1419 10.1016/j.chemosphere.2008.11.031 19108870

[B14] Ma R Sassoon DA PCBs exert an estrogenic effect through repression of the Wnt7a signaling pathway in the female reproductive tract Environ Health Perspect 2006 114 898 904 10.1289/ehp.8748 16759992 PMC1480489

[B15] Toft G Hagmar L Giwercman A Bonde JP Epidemiological evidence on reproductive effects of persistent organochlorines in humans Reprod Toxicol 2004 19 5 26 10.1016/j.reprotox.2004.05.006 15336708

[B16] James WH Hormonal control of sex ratio J Theor Biol 1986 118 427 441 10.1016/S0022-5193(86)80163-1 3713218

[B17] James WH Further support for the hypothesis that parental hormone levels around the time of conception are associated with human sex ratios at birth J Biosoc Sci 2008 40 855 861 10.1017/S0021932008002794 18471338

[B18] James WH Evidence that mammalian sex ratios at birth are partially controlled by parental hormone levels around the time of conception J Endocrinol 2008 198 3 15 10.1677/JOE-07-0446 18577567

[B19] Grech V Vassallo-Agius P Savona-Ventura C Secular trends in sex ratios at birth in North America and Europe over the second half of the 20th century J Epidemiol Community Health 2003 57 612 615 10.1136/jech.57.8.612 12883068 PMC1732531

[B20] Garfinkel J Selvin S A multivariate analysis of the relationship between parental age and birth order and the human secondary sex ratio J Biosoc Sci 1976 8 113 121 10.1017/S0021932000010543 956205

[B21] Ruder A Paternal-age and birth-order effect on the human secondary sex ratio Am J Hum Genet 1985 37 362 372 3985011 PMC1684568

[B22] Teitelbaum MS Mantel N Stark CR Limited dependence of the human sex ratio on birth order and parental ages Am J Hum Genet 1971 23 271 280 5089843 PMC1706718

[B23] Beratis NG Asimacopoulou A Varvarigou A Association of secondary sex ratio with smoking and parity Fertil Steril 2008 89 662 667 10.1016/j.fertnstert.2007.03.034 17517408

[B24] Erickson JD The secondary sex ratio in the United States 1969-71: association with race, parental ages, birth order, paternal education and legitimacy Ann Hum Genet 1976 40 205 212 10.1111/j.1469-1809.1976.tb00182.x 1015815

[B25] Jacobsen R Parental ages and the secondary sex ratio Hum Reprod 2001 16 2244 10.1093/humrep/16.10.2244 11574526

[B26] James WH The variations of human sex ratio at birth with time of conception within the cycle, coital rate around the time of conception, duration of time taken to achieve conception, and duration of gestation: a synthesis J Theor Biol 2008 255 199 204 10.1016/j.jtbi.2008.07.016 18687340

[B27] Kankova S Kodym P Frynta D Vavrinova R Kubena A Flegr J Influence of latent toxoplasmosis on the secondary sex ratio in mice Parasitology 2007 134 1709 1717 10.1017/S0031182007003253 17651529

[B28] Kankova S Sulc J Nouzova K Fajfrlik K Frynta D Flegr J Women infected with parasite Toxoplasma have more sons Naturwissenschaften 2007 94 122 127 10.1007/s00114-006-0166-2 17028886

[B29] Chahnazarian A Blumberg BS London WT Hepatitis B and the sex ratio at birth: a comparative analysis of four populations J Biosoc Sci 1988 20 357 370 10.1017/S0021932000006696 3215916

[B30] del Rio Gomez I Marshall T Tsai P Shao YS Guo YL Number of boys born to men exposed to polychlorinated byphenyls Lancet 2002 360 143 144 10.1016/S0140-6736(02)09386-8 12126828

[B31] Yoshimura T Kaneko S Hayabuchi H Sex ratio in offspring of those affected by dioxin and dioxin-like compounds: the Yusho, Seveso, and Yucheng incidents Occup Environ Med 2001 58 540 541 10.1136/oem.58.8.540 11452050 PMC1740172

[B32] Taylor KC Jackson LW Lynch CD Kostyniak PJ Buck Louis GM Preconception maternal polychlorinated biphenyl concentrations and the secondary sex ratio Environ Res 2007 103 99 105 10.1016/j.envres.2006.04.009 16780830

[B33] Weisskopf MG Anderson HA Hanrahan LP Decreased sex ratio following maternal exposure to polychlorinated biphenyls from contaminated Great Lakes sport-caught fish: a retrospective cohort study Environ Health 2003 2 2 10.1186/1476-069X-2-2 12694628 PMC153540

[B34] Hopf NB Waters MA Ruder AM Cumulative exposure estimates for polychlorinated biphenyls using a job-exposure matrix Chemosphere 2009 76 185 193 10.1016/j.chemosphere.2009.03.058 19394668

[B35] Brown DP Jones M Mortality and industrial hygiene study of workers exposed to polychlorinated biphenyls Arch Environ Health 1981 36 120 129 6787990 10.1080/00039896.1981.10667615

[B36] Sinks T Steele G Smith AB Watkins K Shults RA Mortality among workers exposed to polychlorinated biphenyls Am J Epidemiol 1992 136 389 398 1415158 10.1093/oxfordjournals.aje.a116511

[B37] Smith AH Fisher DO Pearce N Chapman CJ Congenital defects and miscarriages among New Zealand 2, 4, 5-T sprayers Arch Environ Health 1982 37 197 200 7114899 10.1080/00039896.1982.10667564

[B38] Brown DP Mortality of workers exposed to polychlorinated biphenyls--an update Arch Environ Health 1987 42 333 339 10.1080/00039896.1987.9934355 3125795

[B39] Prince MM Hein MJ Ruder AM Waters MA Laber PA Whelan EA Update: cohort mortality study of workers highly exposed to polychlorinated biphenyls (PCBs) during the manufacture of electrical capacitors, 1940-1998 Environ Health 2006 5 13 10.1186/1476-069X-5-13 16716225 PMC1524943

[B40] Prince MM Ruder AM Hein MJ Waters MA Whelan EA Nilsen N Ward EM Schnorr TM Laber PA Davis-King KE Mortality and exposure response among 14,458 electrical capacitor manufacturing workers exposed to polychlorinated biphenyls (PCBs) Environ Health Perspect 2006 114 1508 1514 10.1289/ehp.9175 17035134 PMC1626402

[B41] Ruder AM Hein MJ Nilsen N Waters MA Laber P Davis-King K Prince MM Whelan E Mortality among workers exposed to polychlorinated biphenyls (PCBs) in an electrical capacitor manufacturing plant in Indiana: an update Environ Health Perspect 2006 114 18 23 10.1289/ehp.8253 16393652 PMC1332650

[B42] Steenland K Hein MJ Cassinelli RT Prince MM Nilsen NB Whelan EA Waters MA Ruder AM Schnorr TM Polychlorinated biphenyls and neurodegenerative disease mortality in an occupational cohort Epidemiology 2006 17 8 13 10.1097/01.ede.0000190707.51536.2b 16357589

[B43] Silver SR Whelan EA Deddens JA Steenland NK Hopf NB Waters MA Ruder AM Prince MM Yong LC Hein MJ Ward EM Occupational exposure to polychlorinated biphenyls and risk of breast cancer Environ Health Perspect 2009 117 276 282 19270799 10.1289/ehp.11774 PMC2649231

[B44] Nilsen NB Waters MA Prince MM Zivkovich ZE Ruder AM Workers Exposed to PCBs in a Capacitor Manufacturing Plant (Plant 1; 1948-1977). Cincinnati 2004 [*NIOSH*: NY, Fort Edwards, Hudson Falls]

[B45] Nilsen NB Waters MA Prince MM Zivkovich ZE Ruder AM Workers Exposed to PCBs in a Capacitor Manufacturing Plant (Plant 2; 1938-1977). Cincinnati 2004 [*NIOSH*: MA, New Bedford]

[B46] Hopf NB Waters MA Development of a retrospective job exposure matrix for PCB-exposed workers in capacitor manufacturing J Occup Health 2010 52 1 99 208 20467200 10.1539/joh.l9151

[B47] Vatten LJ Skjaerven R Offspring sex and pregnancy outcome by length of gestation Early Hum Dev 2004 76 47 54 10.1016/j.earlhumdev.2003.10.006 14729162

[B48] Brettell R Yeh PS Impey LW Examination of the association between male gender and preterm delivery Eur J Obstet Gynecol Reprod Biol 2008 141 123 126 10.1016/j.ejogrb.2008.07.030 18783867

[B49] Verner MA Charbonneau M Lopez-Carrillo L Haddad S Physiologically based pharmacokinetic modeling of persistent organic pollutants for lifetime exposure assessment: a new tool in breast cancer epidemiologic studies Environ Health Perspect 2008 116 886 892 10.1289/ehp.10917 18629310 PMC2453156

[B50] Zeitlin J Ancel PY Larroque B Kaminski M Fetal sex and indicated very preterm birth: results of the EPIPAGE study Am J Obstet Gynecol 2004 190 1322 1325 10.1016/j.ajog.2003.10.703 15167836

[B51] Kleinbaum DG Kupper LL Muller KE Nizam A Applied Regression Analysis and Other Multivariable Methods 1998 3 Pacific Grove, CA: Duxbury Press - Brooks/Cole Publishing Co

[B52] Terrell ML Berzen AK Small CM Cameron LL Wirth JJ Marcus M A cohort study of the association between secondary sex ratio and parental exposure to polybrominated biphenyl (PBB) and polychlorinated biphenyl (PCB) Environ Health 2009 8 35 10.1186/1476-069X-8-35 19682390 PMC2794027

[B53] Hertz-Picciotto I Jusko TA Willman EJ Baker RJ Keller JA Teplin SW Charles MJ A cohort study of in utero polychlorinated biphenyl (PCB) exposures in relation to secondary sex ratio Environ Health 2008 7 37 10.1186/1476-069X-7-37 18627595 PMC2483969

[B54] Wolff MS Fischbein A Selikoff IJ Changes in PCB serum concentrations among capacitor manufacturing workers Environ Res 1992 59 202 216 10.1016/S0013-9351(05)80240-3 1425510

[B55] Smith AB Schloemer J Lowry LK Smallwood AW Ligo RN Tanaka S Stringer W Jones M Hervin R Glueck CJ Metabolic and health consequences of occupational exposure to polychlorinated biphenyls Br J Ind Med 1982 39 361 369 6128023 10.1136/oem.39.4.361 PMC1009067

[B56] Rogan WJ Gladen BC Guo YL Hsu CC Sex ratio after exposure to dioxin-like chemicals in Taiwan Lancet 1999 353 206 207 10.1016/S0140-6736(05)77215-9 9923880

[B57] Guo YL Lambert GH Hsu CC Hsu MM Yucheng: health effects of prenatal exposure to polychlorinated biphenyls and dibenzofurans Int Arch Occup Environ Health 2004 77 153 158 10.1007/s00420-003-0487-9 14963712

[B58] Wise LA Palmer JR Hatch EE Troisi R Titus-Ernstoff L Herbst AL Kaufman R Noller KL Hoover RN Secondary sex ratio among women exposed to diethylstilbestrol in utero Environ Health Perspect 2007 115 1314 1319 10.1289/ehp.10246 17805421 PMC1964903

[B59] Wise LA Titus-Ernstoff L Palmer JR Hoover RN Hatch EE Perez KM Strohsnitter WC Kaufman R Anderson D Troisi R Time to pregnancy and secondary sex ratio in men exposed prenatally to diethylstilbestrol Am J Epidemiol 2007 166 765 774 10.1093/aje/kwm136 17596265

[B60] Kodama H Ota H Transfer of polychlorinated biphenyls to infants from their mothers Arch Environ Health 1980 35 95 100 6768342

[B61] Stewart PA Carel R Schairer C Blair A Comparison of industrial hygienists' exposure evaluations for an epidemiologic study Scand J Work Environ Health 2000 26 44 51 10744177 10.5271/sjweh.509

[B62] Tiido T Rignell-Hydbom A Jonsson BA Giwercman YL Pedersen HS Wojtyniak B Ludwicki JK Lesovoy V Zvyezday V Spano M Impact of PCB and p,p'-DDE contaminants on human sperm Y:X chromosome ratio: studies in three European populations and the Inuit population in Greenland Environ Health Perspect 2006 114 718 724 10.1289/ehp.8668 16675426 PMC1459925

