# Supplementary material for: Maternal exposure to polychlorinated biphenyls and the secondary sex ratio: an occupational cohort study
Source: Environ Health. 2011 Mar 18;10:20. doi: 10.1186/1476-069X-10-20 (PMC3070618; doi:10.1186/1476-069X-10-20)
Supplement: Additional file 1 — Odds of birth of a male infant among women occupationally PCB-exposed and unexposed prior to estimated date of conception, adjusted by plant. This table (adjusted_by_plant.doc), in Microsoft Word 2003, displays the odds of birth of a male infant among women occupationally PCB-exposed and unexposed prior to estimated date of conception, adjusted for plant, mother's date of birth, and maternal age at the birth of her first live-born child. [file 1476-069X-10-20-S1.DOC]

# Additional files

### Additional file 1 – Odds of birth of a male infant among women occupationally PCB-exposed and unexposed prior to estimated date of conception, adjusted by plant

| **Estimated Cumulative PCB exposure^a^, at estimated date of conception** | | **Exposed Women (n)** | **Crude**  **OR (95% CI)** | **Adjusted^b^**  **OR (95% CI)** |
| --- | --- | --- | --- | --- |
| Continuous model (log linear) | |  |  |  |
|  | Per 100,000 increase | 1,506 | 1.00 (0.98, 1.03) | 1.01 (.98, 1.04) |
|  |  |  |  |  |
| Categorical model | |  |  |  |
|  | No exposure | 1,089 | 1.00 (ref) | 1.00 (ref) |
|  | >0 to <27,900 | 377 | 0.94 (0.75, 1.19) | 0.93 (0.72, 1.19) |
|  | 27,900 to <108,140 | 376 | 1.14 (0.90, 1.44) | 1.13 (0.88, 1.47) |
|  | 108,140 to <300,216 | 377 | 0.90 (0.72, 1.14) | 0.94 (0.72, 1.23) |
|  | 300,216+ | 376 | 1.02 (0.81, 1.29) | 1.11 (0.85, 1.46) |

^a^ Cumulative exposure was estimated using the combined inhalation–dermal job exposure matrix

**^b^** Adjusted for the plant, the mother’s date of birth, and age at birth of her first born
